# Monoaminergic System Modulation in Depression and Alzheimer’s Disease: A New Standpoint?

**DOI:** 10.3389/fphar.2019.00483

**Published:** 2019-05-17

**Authors:** Maria Grazia Morgese, Luigia Trabace

**Affiliations:** Department of Clinical and Experimental Medicine, University of Foggia, Foggia, Italy

**Keywords:** Alzheimer’s disease, depression, noradrenaline, serotonin, dopamine, beta amyloid

## Abstract

The prevalence of depression has dramatically increased, and it has been estimated that over 300 million people suffer from depression all over the world. Depression is highly comorbid with many central and peripheral disorders. In this regard, depressive states have been associated with the development of neurological disorders such as Alzheimer’s disease (AD). Accordingly, depression is a risk factor for AD and depressive symptomatology is common in pre-clinical AD, representing an early manifestation of this disease. Neuropsychiatric symptoms may represent prodromal symptoms of dementia deriving from neurobiological changes in specific cerebral regions; thus, the search for common biological substrates is becoming an imperative and intriguing field of research. Soluble forms of beta amyloid peptide (Aβ) have been implicated both in the development of early memory deficits and neuropsychiatric symptoms. Indeed, soluble Aβ species have been shown to induce a depressive-like phenotype in AD animal models. Alterations in monoamine content are a common feature of these neuropathologies. Interestingly, serotonergic system modulation has been implicated in alteration of Aβ production. In addition, noradrenaline is considered crucially involved in compensatory mechanisms, leading to increased Aβ degradation *via* several mechanisms, including microglia modulation. In further agreement, antidepressant drugs have also been shown to potentially modulate cognitive symptoms in AD and depression. Thus, the present review summarizes the main knowledge about biological and pathological substrates, such as monoamine and related molecules, commonly involved in AD and depression pathology, thus shading light on new therapeutic approaches.

## Introduction

Many pathologies have been indicated as comorbid with Alzheimer’s diseases (AD) and in particular neuropsychiatric disorders such as depression (Ownby et al., 2006; Sun et al., 2008). Indeed, depression is common in pre-clinical AD and may represent an early manifestation of this disease before the appearance of cognitive impairments (Geerlings et al., 2000; Visser et al., 2000). In this regard, much evidence endorses a strong relationship between depression and AD, so much that this mental illness has been proposed as a risk factor for AD or as a prodromic AD phase (Modrego and Ferrandez, 2004). The amyloid cascade hypothesis postulates that neurodegeneration in AD is related to abnormal accumulation of amyloid beta (Aβ) plaques in various areas of the brain. However, soluble forms of this peptide have been implicated in the development of early memory deficits as well as of neuropsychiatric symptoms (Rowan et al., 2005). Indeed, significant cognitive deficits have been directly attributed to soluble Aβ fragments (Mattson, 2004; Cleary et al., 2005), and increased levels of soluble Aβ oligomers have been linked to synaptic dysfunction (Hardy and Selkoe, 2002; Selkoe and Schenk, 2003). Meanwhile, it has been reported that in depressed patients, Aβ peptide levels are increased (Pomara and Sidtis, 2010). In good agreement, we have previously demonstrated that Aβ, intracerebroventricularly (icv) injected in rats 7 days earlier, evokes a depressive-like profile accompanied by lower cortical serotonin (5-HT) and neurotrophin content (Colaianna et al., 2010). Furthermore, we later reported that such impairment was associated with altered stress response and increased noradrenaline (NA) levels (Morgese et al., 2014, 2015). In addition, in the same model, cognitive impairment was demonstrated either acutely, such as 2 h after Aβ administration, or more enduringly, i.e., 7 days after the peptide central release (Morgese et al., 2014; Tucci et al., 2014; Mhillaj et al., 2018). Although the role of dopamine (DA) was less studied concerning depression and AD, recently, its role has been brought to the fore (Nobili et al., 2017) but is still in need of further evaluation.

The present review is aimed at summarizing the main knowledge related to biological and pathological substrates, such as monoamines and related molecules, commonly involved in AD and depressive pathology, with the scope of shedding light on possible therapeutic approaches.

## Monoamine System in Depression and Alzheimer’s Disease

### Serotonergic System

The treatment of affective disorders is mainly based on the enhancement of the noradrenergic and serotonergic systems through selective or nonselective reuptake inhibitors. Such a pharmacological schedule sinks the roots on the catecholaminergic theory of affective disorders stating the crucial role of lower central NA and 5-HT availability in the insurgence of depression (Mann et al., 1986; Schildkraut, 1995; Mann, 1999). Alterations in these neurotransmitter systems have also been linked to neurodegenerative disorders such as AD. Impairment of the serotonergic system has been reported in the very early stages of AD (Versijpt et al., 2003; Egashira et al., 2005; Kepe et al., 2006), and substantial disruption of the serotonergic system in AD has been postulated according to both clinical and *postmortem* studies (Morgan et al., 1987; Lanctot et al., 2001). In this regard, Aβ in its soluble forms, either monomeric or oligomeric, has been associated with the modulation of these systems. In particular, we have previously found that soluble Aβ injected icv in rats caused a significant reduction in 5-HT at the prefrontal cortex level, without interfering with the physiological functioning of other areas such as the striatum or the nucleus accumbens (Colaianna et al., 2010). These results indicated that the prefrontal cortex is an area highly sensitive to Aβ effects, and this area is also crucially involved in the etiopathogenesis of depressive phenomena. Indeed, impairment of 5-HT neurotransmission in the prefrontal area is central to both depressive disorders (Krishnan and Nestler, 2008) and several neurodegenerative diseases (Mattson, 2004; Egashira et al., 2005). Furthermore, we have more recently individuated the vulnerability of the hippocampal area to the action of exogenous Aβ icv injected. Indeed, we have found that this peptide can reduce 5-HT levels in the hippocampus, and this event is associated with a proinflammatory state and higher rate of activated microglia (Mhillaj et al., 2018). In addition, the treatment with a selective COX-2 inhibitor, such as celecoxib, was able to prevent the reduction in 5-HT levels, thus preventing the Aβ-induced depressive-like behavior and restoring Aβ plasma levels to control (Mhillaj et al., 2018; Morgese et al., 2018a). Accordingly, we have recently demonstrated that environmental factors, such as modified dietary factors, can lead to serotonergic impairment associated with increased levels of Aβ. In particular, we found that deficiency in polyunsaturated fatty acids of the omega 3 family, thus corresponding to a condition linked to a pseudoinflammatory state (Solbrig et al., 2010; Graeber et al., 2011), led to a depressive-like phenotype characterized by reduced 5-HT content and higher Aβ levels (Morgese et al., 2017). Accordingly, an anti-inflammatory diet, such as a diet enriched in omega 3 fatty acids, was able to prevent the reduction in 5-HT caused by Aβ injection, preventing the depressive phenomenon (Bove et al., 2018; Morgese et al., 2018b). Likewise, depressed patients showed higher risk for the development of AD (Kessing and Andersen, 2004). On the other hand, *postmortem* studies performed in AD patients revealed low 5-HT and relative receptor content (Reynolds et al., 1995). An *in vitro* model of familiar AD confirmed these observations, since cells overexpressing APP gene with the Swedish mutations associated with familial AD, indicated an altered sensitivity of the serotonergic system and 5-HT_1B_ receptor subtype in particular (Tajeddinn et al., 2016). Furthermore, in a double transgenic model of early AD, fluoxetine, an antidepressant drug acting as serotonin-selective re-uptake inhibitors (SSRIs), ameliorated the impairment of spatial learning by preventing neuronal loss (Ma et al., 2017) and delayed the cognitive decline associated with synaptic changes (Zhou et al., 2018). Accordingly, clinical evidence revealed that SSRIs significantly improve depressant symptoms and daily activities in AD patients (Werner and Covenas, 2015). This point is very intriguing considering that cognitive decline is recognized also as a clinical feature of depressive state. Interestingly, serotonergic system activation was reported to negatively modulate interstitial Aβ content. Indeed, in transgenic animal models of AD, the enhancing of 5-HT signaling, through the administration of SSRI antidepressants, rapidly reduced Aβ production *in vivo via* activation of extracellular regulated kinase (ERK) and the α-secretase-mediated pathway (Cirrito et al., 2011; Fisher et al., 2016). Indeed, the sequential proteolytic cleavage of amyloid precursor protein (APP) can also occur *via* α-secretase, leading to the production of α-CTF later transformed by γ-secretase into AICD and p3 peptides (Chow et al., 2010). This pathway is recognized as the non-amyloidogenic pathway since APP is cleaved by α-secretase in the Aβ region, yielding to lower Aβ production (Chow et al., 2010). This pathway has been described as neurotrophic and neuroprotective (Chow et al., 2010); therefore, therapeutic strategies steered at pushing APP processing toward α-secretase-mediated derivatives are under the spotlight. Furthermore, a PET imaging study carried out in cognitively normal individuals evidenced lower Aβ accumulation in consequence to increased 5-HT signaling (Sheline et al., 2014), and retrospective analysis on patients under antidepressants further confirmed this finding (Vlassenko et al., 2011). In this regard, we have recently demonstrated that fluoxetine treatment not only could restore 5-HT content in animals centrally injected with Aβ characterized by depressive-like phenotype but also reduced Aβ plasma levels (Schiavone et al., 2017). In further agreement, activation of serotonergic receptors, such as 5-HT_4_, 5-HT_6_, and 5-HT_7_, corresponded to lower Aβ content, whereas the opposite effect was retrieved after simultaneous pharmacological blockade of 5-HT_4_ and 5-HT_7_ (Cho and Hu, 2007; Fisher et al., 2016). 5-HT_4_ partial agonists have been proposed as fast-acting antidepressants (Lucas et al., 2007; Vidal et al., 2014) and have been shown to ameliorate cognitive deficit in anxiety/depressive models (Darcet et al., 2016). In good agreement, pharmacological activation of 5-HT_4_ receptors was shown to enhance short- and long-term memory function (Meneses, 2007), endorsing the hypothesis of a putative role of these drugs for the amelioration of symptomatology of depression in AD. With regard to other receptor subtypes, it has been shown that APP can be released upon activation of 5-HT_2A_ and 5-HT_2C_, and activation of 5-HT_2C_ receptor promotes the expression of neprilysin, a well-characterized Aβ degrading enzymes (Tian et al., 2015). However, it should be considered that both 5-HT_2C_ agonists and antagonists have been evaluated as antidepressants (Cryan and Lucki, 2000; Steardo et al., 2000; Cryan et al., 2005).

As regard to 5-HT_2A_ receptors, genetic polymorphisms have been described in AD patients affected by major depression (Holmes et al., 2003) and, in AD patients, lower binding to these receptors has been identified (Versijpt et al., 2003). In addition, intra-hippocampal injection of Aβ was associated with a significant reduction in 5-HT_2A_ expression (Christensen et al., 2008). However, the effects of the activation of these receptors may vary depending on the cerebral pathway involved. Indeed, 5-HT_2A_ knocked down mice showed an altered phenotype with depressive-like symptoms (Popa et al., 2005), and 5-HT_2A_ antagonists have been evaluated as antidepressants (Zhang and Stackman, 2015); thus, a better understanding would help the developing of targeted compounds. On the other hand, 5-HT_6_ receptors represent a novel therapeutic strategy in AD. Indeed, clinical trial for studying the efficacy and tolerability of the 5-HT_6_ receptor antagonist, SB-742457, in subjects with mild-to-moderate and probable AD, revealed a safe profile and possible utility in improving cognitive symptoms of AD (Maher-Edwards et al., 2010). However, antagonists of these receptor subtypes have been indicated as useful also in the treatment of non-cognitive symptoms associated with AD (Garcia-Alloza et al., 2004). However, despite early positive findings, larger phase-III trials have failed to demonstrate any statistically significant impact on cognition for either idalopirdine or intepirdine, two 5-HT_6_ antagonists, as adjunct to cholinesterase inhibitors. Paradoxically, 5-HT_6_ receptor agonists also hold cognitive enhancing properties (Khoury et al., 2018). Likewise, polymorphism of these receptors has been associated with altered response to antidepressant treatment in major depressive disorder (Lee et al., 2005), although contrasting results have been reported (Wu et al., 2001); hence, further research is warranted.

### Noradrenergic System

The noradrenergic system is also implicated in the etiopathogenesis of both depression and AD. However, it has been recognized that the cause of depression is more complex than just an alteration in the levels of 5-HT and/or NA, being more directly caused by dysfunction in brain areas or neuronal systems modulated by monoamine systems (Delgado and Moreno, 2000). It has been postulated that antidepressants, by enhancing neurotransmission in normal noradrenergic or serotonergic neurons, can restore lost functions in affected brain areas under monoamine control through a time-dependent process (Delgado and Moreno, 2000). Indeed, noradrenergic and serotonergic systems are strictly interconnected and control each other *via* heteroreceptors. In particular, a negative feedback has been hypothesized considering that increased 5-HT levels correspond to NA release, which in turn inhibits further 5-HT release *via* α_2_AR activation (Mongeau et al., 1997). This process is mediated through inhibitory α_2_ receptors (α_2_AR) at 5-HT terminal levels and 5-HT_3_ receptors at NA terminals. Interestingly, increased α_2_AR have been found in *postmortem* brains of depressed patients (Meana et al., 1992; Ordway et al., 1994), and a theory of α_2_AR supersensitivity in depression was postulated early on Charney et al., 1981. In this regard, increased α2-adrenoceptor density was retrieved in most regions of a rat model of depression, such as the flinders sensitive rat (Lillethorup et al., 2015) and in patients with depressive disorders (Cottingham and Wang, 2012). Interestingly, it has been postulated that tricyclic compounds can bind α2AR, thus functioning as arrestin-based ligands, and such an effect can explain their antidepressant property (Cottingham et al., 2015). Βeta-arrestins are a small family of regulators of G protein-coupled receptors that regulate desensitization, internalization along, and initiation of their own signaling of such receptors (Jiang et al., 2013). Long-term activation of these receptors causes endocytosis and downregulation through the recruitment of α_2_AR/arrestin complex (Cottingham et al., 2015). The NA system is deeply affected also in neurodegeneration and in early AD (Haglund et al., 2006). Indeed, α2A adrenergic receptors modulate APP endocytic sorting and promote Aβ generation through disrupting APP interaction with a vacuolar protein sorting (Vps10) family protein, a family of receptors that plays a decisive role in controlling the outcome of APP proteolytic processing (Chen et al., 2014). In addition, this study pointed to the use of α2A antagonists as a new direction for AD treatment. In this light, another putative target for the generation of novel AD treatments is targeting β-arrestin. Indeed, increased β-arrestin 1 levels were shown in a transgenic animal model of AD as well as in *postmortem* study (Liu et al., 2013). In keeping in mind a parallel route for depression and AD, β-arrestin signaling has also been associated with antidepressant properties of drugs (Golan et al., 2013). Overexpression of β-arrestin 2 was associated with increased Aβ production. In particular, experimental conditions able to silence the β-arrestin 2 gene corresponded to Aβ rate of production by regulating γ-secretase activity (Thathiah et al., 2013). Accordingly, Pontrello et al. found that the loss of dendritic spine in hippocampal neurons caused by Aβ was prevented by deleting β-arrestin-2 (Pontrello et al., 2012). On the other hand, polymorphisms in the gene encoding for β2 adrenergic receptor have been associated with an increased risk of developing sporadic late onset AD (Yu et al., 2008), while alterations in β adrenergic receptors were reported in depressed patients (Mann et al., 1986). Indeed, much evidence indicates that activation of these receptors yield to antidepressant effects (Overstreet et al., 2008; Gu et al., 2012). Nonetheless, Aβ interacts with the noradrenergic system directly binding to β-adrenergic receptors (Igbavboa et al., 2006; Wang et al., 2011). Aβ may cause desensitization and subsequently internalization of β2 adrenergic receptors in prefrontal cortical neurons (Wang et al., 2011). Furthermore, β2 adrenergic receptor activation mediates phosphorylation of tau after Aβ exposure both *in vivo* and *in vitro* (Wang et al., 2013). On the other hand, we have found that central icv injection of Aβ increases noradrenergic tone after either 2 h or after 7 days from the central injection, probably reflecting a neuroprotective phenomenon (Morgese et al., 2014, 2015), considering that, NA is protective against neuroinflammatory processes. Accordingly, NA is able to modulate glial activation, and pharmacological strategies finalized to increase NA are considered a valid approach for neurodegenerative diseases (Braun et al., 2014). *In vitro* studies have evidenced that neuroprotective effects of noradrenergic locus coeruleus (LC) afferents against Aβ rely on the stimulation of neurotrophic NGF and BDNF autocrine or paracrine loops *via* beta adrenoceptor activation of the cAMP response element binding protein pathway (Counts and Mufson, 2010; Liu et al., 2015). After Aβ exposure, lower NA concentrations in LC projecting areas facilitate the inflammatory reaction of microglial cells, thus impairing microglial migration and phagocytosis, ultimately decreasing Aβ clearance (Heneka et al., 2010). Accordingly, progression of AD is paralleled by the loss of noradrenergic function in LC (Kelly et al., 2017), indicating the crucial role of this system in neurodegeneration.

### Dopaminergic System

As regards the dopaminergic system, impairment of its neurotransmission has been implicated in many diseases including depression (Schmidt et al., 2001), and several pre-clinical studies have indicated the involvement of dopaminergic, either D1, D2, or D3, in antidepressant effects (Pytka et al., 2016). In good agreement, it has been shown that pure dopaminergic drugs, such as pramipexole, DA precursors, and DA reuptake inhibitors, show therapeutic efficacy in depression (El Mansari et al., 2010; Belujon and Grace, 2017). In addition, neurodegenerative diseases associated with the loss of dopaminergic function, such as Parkinson’s or Huntington’s diseases, have high comorbidities with depression and anxiety (Dale et al., 2016; Schrag and Taddei, 2017; Smeltere et al., 2017).

Concerning AD, it was shown that prefrontal cortical and hippocampal areas showed lower DA receptor expression (Kemppainen et al., 2003; Kumar and Patel, 2007). Interestingly accumbal expression of D2-like receptors, dopaminergic transporter, and tyrosine hydroxylase enzyme was found altered in AD brains (Rinne et al., 1986; Allard et al., 1990; Murray et al., 1995; Joyce et al., 1997). Imaging studies evidenced atrophy of this area in a cohort of AD patients (Pievani et al., 2013). Aβ administration disrupts the cholinergic control of DA release, particularly in the nucleus accumbens (Preda et al., 2008), but we also reported a blunting of DA release in the prefrontal cortex of rat after icv injection of the peptide (Trabace et al., 2007). In addition, the increase in DAnergic tone has been proposed as a possible therapeutic strategy for AD, considering that dopaminergic dysfunction plays a pathogenic role in cognitive decline (Martorana et al., 2009, 2013; Koch et al., 2014; Martorana and Koch, 2014). Furthermore, selective DAnergic neuronal degeneration in ventral tegmental area was demonstrated in AD transgenic mice at pre-plaque stages, suggesting that lower hippocampal and accumbal DA outflow correlate to memory deficits and dysfunction of reward processing (Nobili et al., 2017).

## Conclusions

It has been reported that depressed individuals are nearly twice as likely to develop dementia, often in the form of AD, compared with non-depressed individuals. Unfortunately, few pharmacological tools are available for dementia; thus, the need for novel therapeutic strategies is very compelling. Future studies aimed at elucidating the mechanisms through which drugs modulating monoamine release may prove helpful in individuating novel strategy for slowing down cognitive impairment in pre-clinical AD phase, often associated with mood alterations, taking into account their effects on Aβ production/clearance, aggregation status, and neuroinflammatory-induced pathways. Furthermore, some of these molecules are already commercialized; thus, such a novel potential therapeutic approach for AD treatment may become rapidly clinically suitable.

## Author Contributions

MM and LT helped in study design, drafting, revising, and accepting of the final version of the manuscript.

### Conflict of Interest Statement

The authors declare that the research was conducted in the absence of any commercial or financial relationships that could be construed as a potential conflict of interest.

## References

[ref1] Allard P. Alafuzoff I. Carlsson A. Eriksson K. Ericson E. Gottfries C. G. (1990). Loss of dopamine uptake sites labeled with [3H]GBR-12935 in Alzheimer’s disease. Eur. Neurol. 30, 181–185. 2209670 10.1159/000117341

[ref2] Belujon P. Grace A. A. (2017). Dopamine system dysregulation in major depressive disorders. Int. J. Neuropsychopharmacol. 20, 1036–1046. 10.1093/ijnp/pyx056, PMID: 29106542 PMC5716179

[ref3] Bove M. Mhillaj E. Tucci P. Giardino I. Schiavone S. Morgese M. G.. (2018). Effects of n-3 PUFA enriched and n-3 PUFA deficient diets in naïve and Aβ-treated female rats. Biochem. Pharmacol. 155, 326–335. 10.1016/j.bcp.2018.07.017, PMID: 30028991

[ref4] Braun D. Madrigal J. L. Feinstein D. L. (2014). Noradrenergic regulation of glial activation: molecular mechanisms and therapeutic implications. Curr. Neuropharmacol. 12, 342–352. 10.2174/1570159X12666140828220938, PMID: 25342942 PMC4207074

[ref5] Charney D. S. Menkes D. B. Heninger G. R. (1981). Receptor sensitivity and the mechanism of action of antidepressant treatment. Implications for the etiology and therapy of depression. Arch. Gen. Psychiatry 38, 1160–1180. 10.1001/archpsyc.1981.01780350094011, PMID: 6271089

[ref6] Chen Y. Peng Y. Che P. Gannon M. Liu Y. Li L. (2014). Alpha(2A) adrenergic receptor promotes amyloidogenesis through disrupting APP-SorLA interaction. Proc. Natl. Acad. Sci. USA 111, 17296–17301. 10.1073/pnas.1409513111 25404298 PMC4260556

[ref7] Cho S. Hu Y. (2007). Activation of 5-HT4 receptors inhibits secretion of beta-amyloid peptides and increases neuronal survival. Exp. Neurol. 203, 274–278. 10.1016/j.expneurol.2006.07.021, PMID: 16978609

[ref8] Chow V. W. Mattson M. P. Wong P. C. Gleichmann M. (2010). An overview of APP processing enzymes and products. NeuroMolecular Med. 12, 1–12. 10.1007/s12017-009-8104-z 20232515 PMC2889200

[ref9] Christensen D. Z. Kraus S. L. Flohr A. Cotel M. C. Wirths O. Bayer T. A. (2008). Transient intraneuronal A beta rather than extracellular plaque pathology correlates with neuron loss in the frontal cortex of APP/PS1KI mice. Acta Neuropathol. 116, 647–655. 10.1007/s00401-008-0451-6, PMID: 18974993

[ref10] Cirrito J. R. Disabato B. M. Restivo J. L. Verges D. K. Goebel W. D. Sathyan A.. (2011). Serotonin signaling is associated with lower amyloid-beta levels and plaques in transgenic mice and humans. Proc. Natl. Acad. Sci. USA 108, 14968–14973. 10.1073/pnas.1107411108, PMID: 21873225 PMC3169155

[ref11] Cleary J. P. Walsh D. M. Hofmeister J. J. Shankar G. M. Kuskowski M. A. Selkoe D. J.. (2005). Natural oligomers of the amyloid-beta protein specifically disrupt cognitive function. Nat. Neurosci. 8, 79–84. 10.1038/nn1372, PMID: 15608634

[ref12] Colaianna M. Tucci P. Zotti M. Morgese M. G. Schiavone S. Govoni S.. (2010). Soluble beta amyloid(1-42): a critical player in producing behavioural and biochemical changes evoking depressive-related state? Br. J. Pharmacol. 159, 1704–1715. 10.1111/j.1476-5381.2010.00669.x, PMID: 20218978 PMC2925493

[ref13] Cottingham C. Ferryman C. J. Wang Q. (2015). Alpha2 adrenergic receptor trafficking as a therapeutic target in antidepressant drug action. Prog. Mol. Biol. Transl. Sci. 132, 207–225. 10.1016/bs.pmbts.2015.03.007, PMID: 26055060

[ref14] Cottingham C. Wang Q. (2012). Alpha2 adrenergic receptor dysregulation in depressive disorders: implications for the neurobiology of depression and antidepressant therapy. Neurosci. Biobehav. Rev. 36, 2214–2225. 10.1016/j.neubiorev.2012.07.011, PMID: 22910678 PMC3508310

[ref15] Counts S. E. Mufson E. J. (2010). Noradrenaline activation of neurotrophic pathways protects against neuronal amyloid toxicity. J. Neurochem. 113, 649–660. 10.1111/j.1471-4159.2010.06622.x, PMID: 20132474 PMC2913691

[ref16] Cryan J. F. Lucki I. (2000). Antidepressant-like behavioral effects mediated by 5-Hydroxytryptamine(2C) receptors. J. Pharmacol. Exp. Ther. 295, 1120–1126. PMID: 11082448

[ref17] Cryan J. F. Valentino R. J. Lucki I. (2005). Assessing substrates underlying the behavioral effects of antidepressants using the modified rat forced swimming test. Neurosci. Biobehav. Rev. 29, 547–569. 10.1016/j.neubiorev.2005.03.008, PMID: 15893822

[ref18] Dale M. Maltby J. Shimozaki S. Cramp R. Rickards H. REGISTRY Investigators of the European Huntington’s Disease Network (2016). Disease stage, but not sex, predicts depression and psychological distress in Huntington’s disease: a European population study. J. Psychosom. Res. 80, 17–22. 10.1016/j.jpsychores.2015.11.003, PMID: 26721543

[ref19] Darcet F. Gardier A. M. David D. J. Guilloux J. P. (2016). Chronic 5-HT4 receptor agonist treatment restores learning and memory deficits in a neuroendocrine mouse model of anxiety/depression. Neurosci. Lett. 616, 197–203. 10.1016/j.neulet.2016.01.055, PMID: 26850572

[ref20] Delgado P. L. Moreno F. A. (2000). Role of norepinephrine in depression. J. Clin. Psychiatry. 61(Suppl. 1), 5–12. 10703757

[ref21] Egashira N. Iwasaki K. Takashima A. Watanabe T. Kawabe H. Matsuda T.. (2005). Altered depression-related behavior and neurochemical changes in serotonergic neurons in mutant R406W human tau transgenic mice. Brain Res. 1059, 7–12. 10.1016/j.brainres.2005.08.004, PMID: 16182262

[ref22] El Mansari M. Guiard B. P. Chernoloz O. Ghanbari R. Katz N. Blier P. (2010). Relevance of norepinephrine-dopamine interactions in the treatment of major depressive disorder. CNS Neurosci. Ther. 16, e1–e17. 10.1111/j.1755-5949.2010.00146.x, PMID: 20406250 PMC2904493

[ref23] Fisher J. R. Wallace C. E. Tripoli D. L. Sheline Y. I. Cirrito J. R. (2016). Redundant Gs-coupled serotonin receptors regulate amyloid-beta metabolism in vivo. Mol. Neurodegener. 11:45. 10.1186/s13024-016-0112-5, PMID: 27315796 PMC4912779

[ref24] Garcia-Alloza M. Hirst W. D. Chen C. P. Lasheras B. Francis P. T. Ramirez M. J. (2004). Differential involvement of 5-HT(1B/1D) and 5-HT6 receptors in cognitive and non-cognitive symptoms in Alzheimer’s disease. Neuropsychopharmacology 29, 410–416. 10.1038/sj.npp.1300330, PMID: 14571255

[ref25] Geerlings M. I. Schoevers R. A. Beekman A. T. Jonker C. Deeg D. J. Schmand B.. (2000). Depression and risk of cognitive decline and Alzheimer’s disease. Results of two prospective community-based studies in The Netherlands. Br. J. Psychiatry 176, 568–575. 10.1192/bjp.176.6.568, PMID: 10974964

[ref26] Golan M. Schreiber G. Avissar S. (2013). Antidepressant-induced differential ubiquitination of beta-arrestins 1 and 2 in mononuclear leucocytes of patients with depression. Int. J. Neuropsychopharmacol. 16, 1745–1754. 10.1017/S1461145713000291, PMID: 23672745

[ref27] Graeber M. B. Li W. Rodriguez M. L. (2011). Role of microglia in CNS inflammation. FEBS Lett. 585, 3798–3805. 10.1016/j.febslet.2011.08.033 21889505

[ref28] Gu Y. Schupf N. Cosentino S. A. Luchsinger J. A. Scarmeas N. (2012). Nutrient intake and plasma beta-amyloid. Neurology 78, 1832–1840. 10.1212/WNL.0b013e318258f7c2 22551728 PMC3369517

[ref29] Haglund M. Sjobeck M. Englund E. (2006). Locus ceruleus degeneration is ubiquitous in Alzheimer’s disease: possible implications for diagnosis and treatment. Neuropathology 26, 528–532. 10.1111/j.1440-1789.2006.00725.x, PMID: 17203588

[ref30] Hardy J. Selkoe D. J. (2002). The amyloid hypothesis of Alzheimer’s disease: progress and problems on the road to therapeutics. Science 297, 353–356. 10.1126/science.1072994, PMID: 12130773

[ref31] Heneka M. T. Nadrigny F. Regen T. Martinez-Hernandez A. Dumitrescu-Ozimek L. Terwel D. (2010). Locus ceruleus controls Alzheimer’s disease pathology by modulating microglial functions through norepinephrine. Proc. Natl. Acad. Sci. USA 107, 6058–6063. 10.1073/pnas.0909586107 20231476 PMC2851853

[ref32] Holmes C. Arranz M. Collier D. Powell J. Lovestone S. (2003). Depression in Alzheimer’s disease: the effect of serotonin receptor gene variation. Am. J. Med. Genet. B Neuropsychiatr. Genet. 119B, 40–43. 10.1002/ajmg.b.10068, PMID: 12707936

[ref33] Igbavboa U. Johnson-Anuna L. N. Rossello X. Butterick T. A. Sun G. Y. Wood W. G. (2006). Amyloid beta-protein1-42 increases cAMP and apolipoprotein E levels which are inhibited by beta1 and beta2-adrenergic receptor antagonists in mouse primary astrocytes. Neuroscience 142, 655–660. 10.1016/j.neuroscience.2006.06.056, PMID: 16904834

[ref34] Jiang Z. Cowell R. M. Nakazawa K. (2013). Convergence of genetic and environmental factors on parvalbumin-positive interneurons in schizophrenia. Front. Behav. Neurosci. 7:116. 10.3389/fnbeh.2013.00116 24027504 PMC3759852

[ref35] Joyce J. N. Smutzer G. Whitty C. J. Myers A. Bannon M. J. (1997). Differential modification of dopamine transporter and tyrosine hydroxylase mRNAs in midbrain of subjects with Parkinson’s, Alzheimer’s with parkinsonism, and Alzheimer’s disease. Mov. Disord. 12, 885–897. 10.1002/mds.870120609, PMID: 9399211

[ref36] Kelly S. C. He B. Perez S. E. Ginsberg S. D. Mufson E. J. Counts S. E. (2017). Locus coeruleus cellular and molecular pathology during the progression of Alzheimer’s disease. Acta Neuropathol. Commun. 5:8. 10.1186/s40478-017-0411-2, PMID: 28109312 PMC5251221

[ref37] Kemppainen N. Laine M. Laakso M. P. Kaasinen V. Nagren K. Vahlberg T.. (2003). Hippocampal dopamine D2 receptors correlate with memory functions in Alzheimer’s disease. Eur. J. Neurosci. 18, 149–154. 10.1046/j.1460-9568.2003.02716.x, PMID: 12859348

[ref38] Kepe V. Barrio J. R. Huang S. C. Ercoli L. Siddarth P. Shoghi-Jadid K. (2006). Serotonin 1A receptors in the living brain of Alzheimer’s disease patients. Proc. Natl. Acad. Sci. USA 103, 702–707. 10.1073/pnas.0510237103 16407119 PMC1334681

[ref39] Kessing L. V. Andersen P. K. (2004). Does the risk of developing dementia increase with the number of episodes in patients with depressive disorder and in patients with bipolar disorder? J. Neurol. Neurosurg. Psychiatry 75, 1662–1666. 10.1136/jnnp.2003.031773, PMID: 15548477 PMC1738846

[ref40] Khoury R. Grysman N. Gold J. Patel K. Grossberg G. T. (2018). The role of 5 HT6-receptor antagonists in Alzheimer’s disease: an update. Expert Opin. Investig. Drugs 27, 523–533. 10.1080/13543784.2018.1483334 29848076

[ref41] Koch G. Di Lorenzo F. Bonni S. Giacobbe V. Bozzali M. Caltagirone C.. (2014). Dopaminergic modulation of cortical plasticity in Alzheimer’s disease patients. Neuropsychopharmacology 39, 2654–2661. 10.1038/npp.2014.119, PMID: 24859851 PMC4207345

[ref42] Krishnan V. Nestler E. J. (2008). The molecular neurobiology of depression. Nature 455, 894–902. 10.1038/nature07455, PMID: 18923511 PMC2721780

[ref43] Kumar U. Patel S. C. (2007). Immunohistochemical localization of dopamine receptor subtypes (D1R-D5R) in Alzheimer’s disease brain. Brain Res. 1131, 187–196. 10.1016/j.brainres.2006.10.049, PMID: 17182012

[ref44] Lanctot K. L. Herrmann N. Mazzotta P. (2001). Role of serotonin in the behavioral and psychological symptoms of dementia. J. Neuropsychiatr. Clin. Neurosci. 13, 5–21. 10.1176/jnp.13.1.5, PMID: 11207325

[ref45] Lee S. H. Lee K. J. Lee H. J. Ham B. J. Ryu S. H. Lee M. S. (2005). Association between the 5-HT6 receptor C267T polymorphism and response to antidepressant treatment in major depressive disorder. Psychiatry Clin. Neurosci. 59, 140–145. 10.1111/j.1440-1819.2005.01348.x, PMID: 15823158

[ref46] Lillethorup T. P. Iversen P. Wegener G. Doudet D. J. Landau A. M. (2015). Alpha2-adrenoceptor binding in Flinders-sensitive line compared with Flinders-resistant line and Sprague-Dawley rats. Acta Neuropsychiatr. 27, 345–352. 10.1017/neu.2015.24, PMID: 25903810

[ref47] Liu X. Ye K. Weinshenker D. (2015). Norepinephrine protects against amyloid-beta toxicity via TrkB. J. Alzheimers Dis. 44, 251–260. 10.3233/JAD-141062, PMID: 25208620 PMC4714587

[ref48] Liu X. Zhao X. Zeng X. Bossers K. Swaab D. F. Zhao J.. (2013). Beta-arrestin1 regulates gamma-secretase complex assembly and modulates amyloid-beta pathology. Cell Res. 23, 351–365. 10.1038/cr.2012.167, PMID: 23208420 PMC3587707

[ref49] Lucas G. Rymar V. V. Du J. Mnie-Filali O. Bisgaard C. Manta S.. (2007). Serotonin(4) (5-HT(4)) receptor agonists are putative antidepressants with a rapid onset of action. Neuron 55, 712–725. 10.1016/j.neuron.2007.07.041, PMID: 17785179

[ref50] Ma J. Gao Y. Jiang L. Chao F. L. Huang W. Zhou C. N.. (2017). Fluoxetine attenuates the impairment of spatial learning ability and prevents neuron loss in middle-aged APPswe/PSEN1dE9 double transgenic Alzheimer’s disease mice. Oncotarget 8, 27676–27692. 10.18632/oncotarget.15398, PMID: 28430602 PMC5438600

[ref51] Maher-Edwards G. Zvartau-Hind M. Hunter A. J. Gold M. Hopton G. Jacobs G.. (2010). Double-blind, controlled phase II study of a 5-HT6 receptor antagonist, SB-742457, in Alzheimer’s disease. Curr. Alzheimer Res. 7, 374–385. 10.2174/156720510791383831, PMID: 20043816

[ref52] Mann J. J. (1999). Role of the serotonergic system in the pathogenesis of major depression and suicidal behavior. Neuropsychopharmacology 21, 99S–105S. 10.1016/S0893-133X(99)00040-8, PMID: 10432495

[ref53] Mann J. J. Mcbride P. A. Stanley M. (1986). Postmortem serotonergic and adrenergic receptor binding to frontal cortex: correlations with suicide. Psychopharmacol. Bull. 22, 647–649. PMID: 3025907

[ref54] Martorana A. Di Lorenzo F. Esposito Z. Lo Giudice T. Bernardi G. Caltagirone C.. (2013). Dopamine D(2)-agonist rotigotine effects on cortical excitability and central cholinergic transmission in Alzheimer’s disease patients. Neuropharmacology 64, 108–113. 10.1016/j.neuropharm.2012.07.015, PMID: 22863599

[ref55] Martorana A. Koch G. (2014). Is dopamine involved in Alzheimer’s disease? Front. Aging Neurosci. 6:252. 10.3389/fnagi.2014.00252 25309431 PMC4174765

[ref56] Martorana A. Mori F. Esposito Z. Kusayanagi H. Monteleone F. Codeca C.. (2009). Dopamine modulates cholinergic cortical excitability in Alzheimer’s disease patients. Neuropsychopharmacology 34, 2323–2328. 10.1038/npp.2009.60, PMID: 19516251

[ref57] Mattson M. P. (2004). Pathways towards and away from Alzheimer’s disease. Nature 430, 631–639. 10.1038/nature02621, PMID: 15295589 PMC3091392

[ref58] Meana J. J. Barturen F. Garcia-Sevilla J. A. (1992). Alpha 2-adrenoceptors in the brain of suicide victims: increased receptor density associated with major depression. Biol. Psychiatry 31, 471–490. 10.1016/0006-3223(92)90259-3, PMID: 1349830

[ref59] Meneses A. (2007). Stimulation of 5-HT1A, 5-HT1B, 5-HT2A/2C, 5-HT3 and 5-HT4 receptors or 5-HT uptake inhibition: short- and long-term memory. Behav. Brain Res. 184, 81–90. 10.1016/j.bbr.2007.06.026, PMID: 17692935

[ref60] Mhillaj E. Morgese M. G. Tucci P. Furiano A. Luongo L. Bove M.. (2018). Celecoxib prevents cognitive impairment and neuroinflammation in soluble amyloid beta-treated rats. Neuroscience 372, 58–73. 10.1016/j.neuroscience.2017.12.046, PMID: 29306052

[ref61] Modrego P. J. Ferrandez J. (2004). Depression in patients with mild cognitive impairment increases the risk of developing dementia of Alzheimer type: a prospective cohort study. Arch. Neurol. 61, 1290–1293. 10.1001/archneur.61.8.1290, PMID: 15313849

[ref62] Mongeau R. Blier P. De Montigny C. (1997). The serotonergic and noradrenergic systems of the hippocampus: their interactions and the effects of antidepressant treatments. Brain Res. Brain Res. Rev. 23, 145–195. 10.1016/S0165-0173(96)00017-3, PMID: 9164669

[ref63] Morgan D. G. May P. C. Finch C. E. (1987). Dopamine and serotonin systems in human and rodent brain: effects of age and neurodegenerative disease. J. Am. Geriatr. Soc. 35, 334–345. 3549845 10.1111/j.1532-5415.1987.tb04641.x

[ref64] Morgese M. G. Colaianna M. Mhillaj E. Zotti M. Schiavone S. D’antonio P.. (2015). Soluble beta amyloid evokes alteration in brain norepinephrine levels: role of nitric oxide and interleukin-1. Front. Neurosci. 9:428. 10.3389/fnins.2015.00428, PMID: 26594145 PMC4633524

[ref65] Morgese M. G. Schiavone S. Bove M. Mhillaj E. Tucci P. Trabace L. (2018a). Sub-chronic celecoxib prevents soluble beta amyloid-induced depressive-like behaviour in rats. J. Affect. Disord. 238, 118–121. 10.1016/j.jad.2018.05.030 29879605

[ref66] Morgese M. G. Schiavone S. Mhillaj E. Bove M. Tucci P. Trabace L. (2018b). N-3 PUFA diet enrichment prevents amyloid beta-induced depressive-like phenotype. Pharmacol. Res. 129, 526–534. 10.1016/j.phrs.2017.11.034 29203442

[ref67] Morgese M. G. Tucci P. Colaianna M. Zotti M. Cuomo V. Schiavone S.. (2014). Modulatory activity of soluble beta amyloid on HPA axis function in rats. Curr. Pharm. Des. 20, 2539–2546. 10.2174/13816128113199990500, PMID: 23859549

[ref68] Morgese M. G. Tucci P. Mhillaj E. Bove M. Schiavone S. Trabace L.. (2017). Lifelong nutritional omega-3 deficiency evokes depressive-like state through soluble beta amyloid. Mol. Neurobiol. 54, 2079–2089. 10.1007/s12035-016-9809-2, PMID: 26924315 PMC5355522

[ref69] Murray A. M. Weihmueller F. B. Marshall J. F. Hurtig H. I. Gottleib G. L. Joyce J. N. (1995). Damage to dopamine systems differs between Parkinson’s disease and Alzheimer’s disease with parkinsonism. Ann. Neurol. 37, 300–312. 10.1002/ana.410370306, PMID: 7695230

[ref70] Nobili A. Latagliata E. C. Viscomi M. T. Cavallucci V. Cutuli D. Giacovazzo G. (2017). Dopamine neuronal loss contributes to memory and reward dysfunction in a model of Alzheimer’s disease. Nat. Commun. 8:14727. 10.1038/ncomms14727 28367951 PMC5382255

[ref71] Ordway G. A. Widdowson P. S. Smith K. S. Halaris A. (1994). Agonist binding to alpha 2-adrenoceptors is elevated in the locus coeruleus from victims of suicide. J. Neurochem. 63, 617–624. PMID: 8035185 10.1046/j.1471-4159.1994.63020617.x

[ref72] Overstreet D. H. Stemmelin J. Griebel G. (2008). Confirmation of antidepressant potential of the selective beta3 adrenoceptor agonist amibegron in an animal model of depression. Pharmacol. Biochem. Behav. 89, 623–626. 10.1016/j.pbb.2008.02.020, PMID: 18358519

[ref73] Ownby R. L. Crocco E. Acevedo A. John V. Loewenstein D. (2006). Depression and risk for Alzheimer disease: systematic review, meta-analysis, and metaregression analysis. Arch. Gen. Psychiatry 63, 530–538. 10.1001/archpsyc.63.5.530, PMID: 16651510 PMC3530614

[ref74] Pievani M. Bocchetta M. Boccardi M. Cavedo E. Bonetti M. Thompson P. M.. (2013). Striatal morphology in early-onset and late-onset alzheimer’s disease: a preliminary study. Neurobiol. Aging 34, 1728–1739. 10.1016/j.neurobiolaging.2013.01.016, PMID: 23428181

[ref75] Pomara N. Sidtis J. J. (2010). Brain neurotoxic amyloid-beta peptides: their potential role in the pathophysiology of depression and as molecular therapeutic targets. Br. J. Pharmacol. 161, 768–770. 10.1111/j.1476-5381.2010.00948.x, PMID: 21105218 PMC2992893

[ref76] Pontrello C. G. Sun M. Y. Lin A. Fiacco T. A. Defea K. A. Ethell I. M. (2012). Cofilin under control of beta-arrestin-2 in NMDA-dependent dendritic spine plasticity, long-term depression (LTD), and learning. Proc. Natl. Acad. Sci. USA 109, E442–E451. 10.1073/pnas.1118803109 22308427 PMC3289389

[ref77] Popa D. Lena C. Fabre V. Prenat C. Gingrich J. Escourrou P.. (2005). Contribution of 5-HT2 receptor subtypes to sleep-wakefulness and respiratory control, and functional adaptations in knock-out mice lacking 5-HT2A receptors. J. Neurosci. 25, 11231–11238. 10.1523/JNEUROSCI.1724-05.2005, PMID: 16339018 PMC6725907

[ref78] Preda S. Govoni S. Lanni C. Racchi M. Mura E. Grilli M.. (2008). Acute beta-amyloid administration disrupts the cholinergic control of dopamine release in the nucleus accumbens. Neuropsychopharmacology 33, 1062–1070. 10.1038/sj.npp.1301485, PMID: 17581530

[ref79] Pytka K. Podkowa K. Rapacz A. Podkowa A. Zmudzka E. Olczyk A.. (2016). The role of serotonergic, adrenergic and dopaminergic receptors in antidepressant-like effect. Pharmacol. Rep. 68, 263–274. 10.1016/j.pharep.2015.08.007, PMID: 26922526

[ref80] Reynolds G. P. Mason S. L. Meldrum A. De Keczer S. Parnes H. Eglen R. M.. (1995). 5-Hydroxytryptamine (5-HT)4 receptors in post mortem human brain tissue: distribution, pharmacology and effects of neurodegenerative diseases. Br. J. Pharmacol. 114, 993–998. 10.1111/j.1476-5381.1995.tb13303.x, PMID: 7780656 PMC1510307

[ref81] Rinne J. O. Sako E. Paljarvi L. Molsa P. K. Rinne U. K. (1986). Brain dopamine D-1 receptors in senile dementia. J. Neurol. Sci. 73, 219–230. 2871136 10.1016/0022-510x(86)90132-2

[ref82] Rowan M. J. Klyubin I. Wang Q. Anwyl R. (2005). Synaptic plasticity disruption by amyloid beta protein: modulation by potential Alzheimer’s disease modifying therapies. Biochem. Soc. Trans. 33, 563–567. 10.1042/BST0330563, PMID: 16042545

[ref83] Schiavone S. Tucci P. Mhillaj E. Bove M. Trabace L. Morgese M. G. (2017). Antidepressant drugs for beta amyloid-induced depression: a new standpoint? Prog. Neuro-Psychopharmacol. Biol. Psychiatry 78, 114–122. 10.1016/j.pnpbp.2017.05.004, PMID: 28499898

[ref84] Schildkraut J. J. (1995). The catecholamine hypothesis of affective disorders: a review of supporting evidence. 1965. J. Neuropsychiatr. Clin. Neurosci. 7, 524–533. discussion: 523-524. 10.1176/jnp.7.4.524 8555758

[ref85] Schmidt K. Nolte-Zenker B. Patzer J. Bauer M. Schmidt L. G. Heinz A. (2001). Psychopathological correlates of reduced dopamine receptor sensitivity in depression, schizophrenia, and opiate and alcohol dependence. Pharmacopsychiatry 34, 66–72. 10.1055/s-2001-15184, PMID: 11302566

[ref86] Schrag A. Taddei R. N. (2017). Depression and anxiety in Parkinson’s disease. Int. Rev. Neurobiol. 133, 623–655. 10.1016/bs.irn.2017.05.024 28802935

[ref87] Selkoe D. J. Schenk D. (2003). Alzheimer’s disease: molecular understanding predicts amyloid-based therapeutics. Annu. Rev. Pharmacol. Toxicol. 43, 545–584. 10.1146/annurev.pharmtox.43.100901.140248, PMID: 12415125

[ref88] Sheline Y. I. West T. Yarasheski K. Swarm R. Jasielec M. S. Fisher J. R.. (2014). An antidepressant decreases CSF Abeta production in healthy individuals and in transgenic AD mice. Sci. Transl. Med. 6:236re234. 10.1126/scitranslmed.3008169, PMID: 24828079 PMC4269372

[ref89] Smeltere L. Kuznecovs V. Erts R. (2017). Depression and social phobia in essential tremor and Parkinson’s disease. Brain Behav. 7:e00781. 10.1002/brb3.781, PMID: 28948077 PMC5607546

[ref90] Solbrig M. V. Fan Y. Hermanowicz N. Morgese M. G. Giuffrida A. (2010). A synthetic cannabinoid agonist promotes oligodendrogliogenesis during viral encephalitis in rats. Exp. Neurol. 226, 231–241. 10.1016/j.expneurol.2010.09.003, PMID: 20832403 PMC2981070

[ref91] Steardo L. Monteleone P. Trabace L. Cannizzaro C. Maj M. Cuomo V. (2000). Serotonergic modulation of rat pineal gland activity: in vivo evidence for a 5-Hydroxytryptamine(2C) receptor involvement. J. Pharmacol. Exp. Ther. 295, 266–273. PMID: 10991989

[ref92] Sun X. Steffens D. C. Au R. Folstein M. Summergrad P. Yee J.. (2008). Amyloid-associated depression: a prodromal depression of Alzheimer disease? Arch. Gen. Psychiatry 65, 542–550. 10.1001/archpsyc.65.5.542, PMID: 18458206 PMC3042807

[ref93] Tajeddinn W. Persson T. Calvo-Garrido J. Seed Ahmed M. Maioli S. Vijayaraghavan S.. (2016). Pharmacological modulations of the serotonergic system in a cell-model of familial Alzheimer’s disease. J. Alzheimers Dis. 53, 349–361. 10.3233/JAD-160046, PMID: 27163814

[ref94] Thathiah A. Horre K. Snellinx A. Vandewyer E. Huang Y. Ciesielska M.. (2013). beta-arrestin 2 regulates Abeta generation and gamma-secretase activity in Alzheimer’s disease. Nat. Med. 19, 43–49. 10.1038/nm.3023, PMID: 23202293

[ref95] Tian X. L. Yu L. H. Li W. Q. Hu Y. Yin M. Wang Z. J. (2015). Activation of 5-HT(2C) receptor promotes the expression of neprilysin in U251 human glioma cells. Cell. Mol. Neurobiol. 35, 425–432. 10.1007/s10571-014-0138-6, PMID: 25452160 PMC11486189

[ref96] Trabace L. Kendrick K. M. Castrignano S. Colaianna M. De Giorgi A. Schiavone S.. (2007). Soluble amyloid beta1-42 reduces dopamine levels in rat prefrontal cortex: relationship to nitric oxide. Neuroscience 147, 652–663. 10.1016/j.neuroscience.2007.04.056, PMID: 17560043

[ref97] Tucci P. Mhillaj E. Morgese M. G. Colaianna M. Zotti M. Schiavone S.. (2014). Memantine prevents memory consolidation failure induced by soluble beta amyloid in rats. Front. Behav. Neurosci. 8:332. 10.3389/fnbeh.2014.00332, PMID: 25285073 PMC4168698

[ref98] Versijpt J. Van Laere K. J. Dumont F. Decoo D. Vandecapelle M. Santens P.. (2003). Imaging of the 5-HT2A system: age-, gender-, and Alzheimer’s disease-related findings. Neurobiol. Aging 24, 553–561. 10.1016/S0197-4580(02)00137-9, PMID: 12714112

[ref99] Vidal R. Castro E. Pilar-Cuellar F. Pascual-Brazo J. Diaz A. Rojo M. L.. (2014). Serotonin 5-HT4 receptors: a new strategy for developing fast acting antidepressants? Curr. Pharm. Des. 20, 3751–3762. 10.2174/13816128113196660734, PMID: 24180399

[ref100] Visser P. J. Verhey F. R. Ponds R. W. Kester A. Jolles J. (2000). Distinction between preclinical Alzheimer’s disease and depression. J. Am. Geriatr. Soc. 48, 479–484. 10.1111/j.1532-5415.2000.tb04992.x 10811539

[ref101] Vlassenko A. G. Mintun M. A. Xiong C. Sheline Y. I. Goate A. M. Benzinger T. L.. (2011). Amyloid-beta plaque growth in cognitively normal adults: longitudinal [11C]Pittsburgh compound B data. Ann. Neurol. 70, 857–861. 10.1002/ana.22608, PMID: 22162065 PMC3243969

[ref102] Wang D. Fu Q. Zhou Y. Xu B. Shi Q. Igwe B.. (2013). Beta2 adrenergic receptor, protein kinase A (PKA) and c-Jun N-terminal kinase (JNK) signaling pathways mediate tau pathology in Alzheimer disease models. J. Biol. Chem. 288, 10298–10307. 10.1074/jbc.M112.415141, PMID: 23430246 PMC3624413

[ref103] Wang D. Yuen E. Y. Zhou Y. Yan Z. Xiang Y. K. (2011). Amyloid beta peptide-(1-42) induces internalization and degradation of beta2 adrenergic receptors in prefrontal cortical neurons. J. Biol. Chem. 286, 31852–31863. 10.1074/jbc.M111.244335, PMID: 21757762 PMC3173113

[ref104] Werner F. M. Covenas R. (2015). Review: classical neurotransmitters and neuropeptides involved in generalized epilepsy in a multi-neurotransmitter system: how to improve the antiepileptic effect? Epilepsy Behav. 71, 124–129. 10.1016/j.yebeh.2015.01.038 25819950

[ref105] Wu W. H. Huo S. J. Cheng C. Y. Hong C. J. Tsai S. J. (2001). Association study of the 5-HT(6) receptor polymorphism (C267T) and symptomatology and antidepressant response in major depressive disorders. Neuropsychobiology 44, 172–175. 10.1159/000054938, PMID: 11702016

[ref106] Yu J. T. Tan L. Ou J. R. Zhu J. X. Liu K. Song J. H.. (2008). Polymorphisms at the beta2-adrenergic receptor gene influence Alzheimer’s disease susceptibility. Brain Res. 1210, 216–222. 10.1016/j.brainres.2008.03.019, PMID: 18423577

[ref107] Zhang G. Stackman R. W. Jr. (2015). The role of serotonin 5-HT2A receptors in memory and cognition. Front. Pharmacol. 6:225. 10.3389/fphar.2015.00225 26500553 PMC4594018

[ref108] Zhou C. N. Chao F. L. Zhang Y. Jiang L. Zhang L. Fan J. H. (2018). Fluoxetine delays the cognitive function decline and synaptic changes in a transgenic mouse model of early Alzheimer’s disease. J. Comp. Neurol. 527, 1378–1387. 10.1002/cne.24616 30592045

